# Physical modulation of mesenchymal stem cell exosomes: A new perspective for regenerative medicine

**DOI:** 10.1111/cpr.13630

**Published:** 2024-03-10

**Authors:** Dan Wu, Xiansheng Zhao, Jiaheng Xie, Ruoyue Yuan, Yue Li, Quyang Yang, Xiujun Cheng, Changyue Wu, Jinyan Wu, Ningwen Zhu

**Affiliations:** ^1^ Department of Dermatology Huashan Hospital, Fudan University Shanghai China; ^2^ Department of Plastic Surgery Xiangya Hospital, Central South University Changsha Hunan China; ^3^ Department of Dermatology Chongzhou People's Hospital Chengdu China; ^4^ Department of Plastic Reconstructive and Burns Surgery, Huashan Hospital, Fudan University Shanghai China

## Abstract

Mesenchymal stem cell‐derived exosomes (MSC‐Exo) offer promising therapeutic potential for various refractory diseases, presenting a novel therapeutic strategy. However, their clinical application encounters several obstacles, including low natural secretion, uncontrolled biological functions and inherent heterogeneity. On the one hand, physical stimuli can mimic the microenvironment dynamics where MSC‐Exo reside. These factors influence not only their secretion but also, significantly, their biological efficacy. Moreover, physical factors can also serve as techniques for engineering exosomes. Therefore, the realm of physical factors assumes a crucial role in modifying MSC‐Exo, ultimately facilitating their clinical translation. This review focuses on the research progress in applying physical factors to MSC‐Exo, encompassing ultrasound, electrical stimulation, light irradiation, intrinsic physical properties, ionizing radiation, magnetic field, mechanical forces and temperature. We also discuss the current status and potential of physical stimuli‐affected MSC‐Exo in clinical applications. Furthermore, we address the limitations of recent studies in this field. Based on this, this review provides novel insights to advance the refinement of MSC‐Exo as a therapeutic approach in regenerative medicine.

## Graphical abstract

Physical factors influenced both their secretion and, significantly, their biological efficacy. Then, physical factors could also serve as techniques for the engineering of exosomes. The realm of physical factors assumes an instrumental role in modifying and upgrading MSC‐Exo, ultimately facilitating their clinical translation.

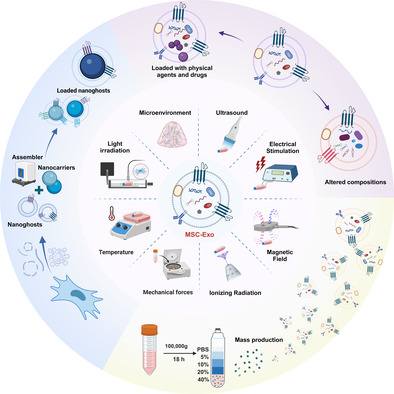

## 1 INTRODUCTION

**FIGURE 1:**
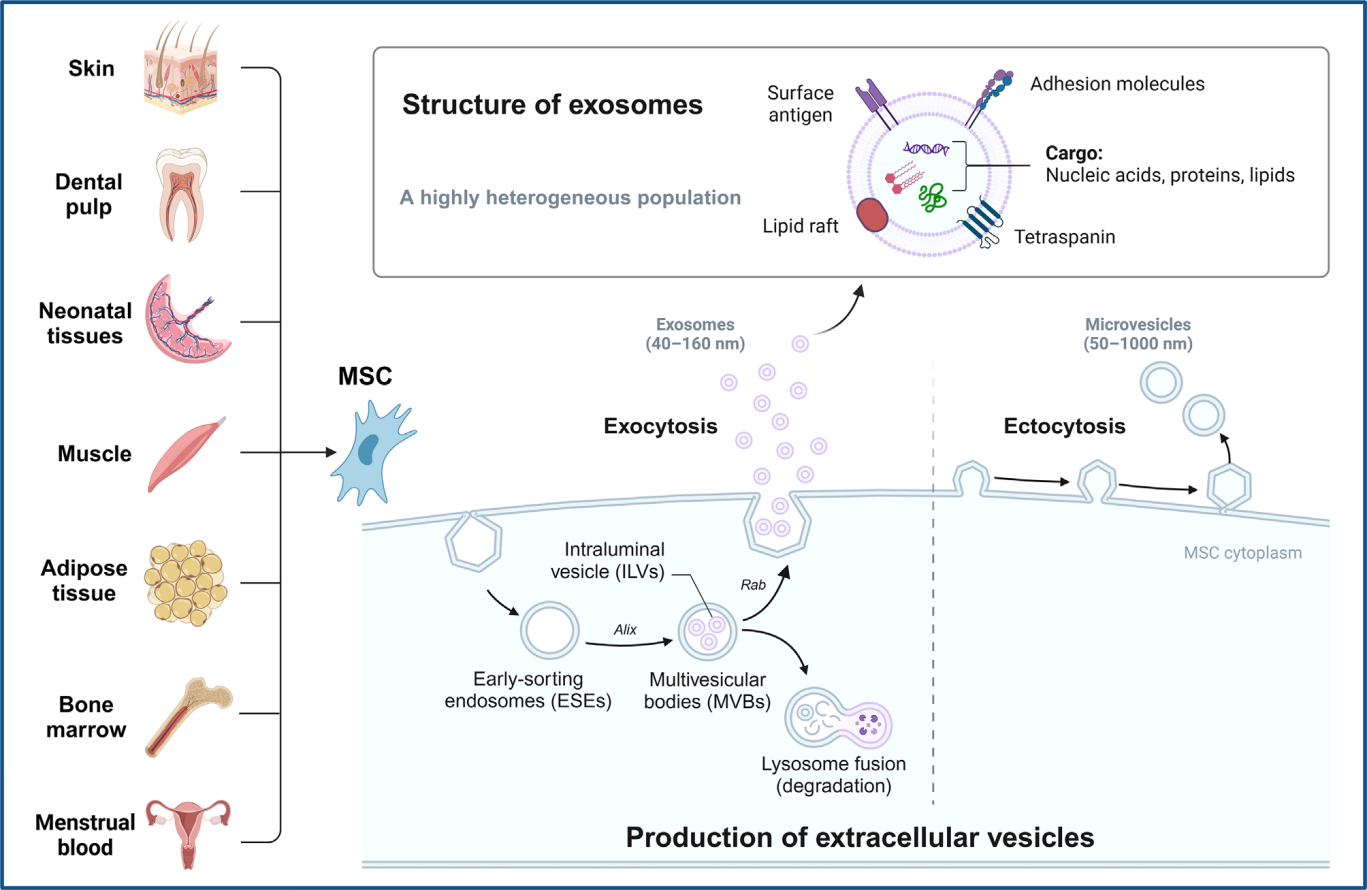
Sources of mesenchymal stem cells and biogenesis of exosomes.

Mesenchymal stem cells (MSCs) represent a heterogeneous population of adult stem cells with the potential for multi‐mesodermal lineage differentiation.^1^ They can be isolated from various tissues, including bone marrow, adipose tissue, neonatal tissues and menstrual blood, among others (as shown in Figure 1).^2^ MSCs are widely recognized for their ability to promote tissue repair and regulate immune response through various mechanisms, such as cell proliferation and differentiation in situ, intercellular interactions and the secretion of paracrine factors.^2^, ^3^, ^4^, ^5^ Notably, the paracrine effects of MSCs, especially those mediated via exosomes have garnered significant attention in recent years. Several studies have highlighted that the therapeutic benefits of MSCs are, in part, attributed to the release of MSC‐derived exosomes (MSC‐Exo), rather than solely relying on their cellular properties within the damaged area.^5^, ^6^, ^7^, ^8^

Exosomes are known as nano‐sized extracellular vesicles containing a variety of components, including bioactive proteins, nucleic acids and lipids.^9^ In general, exosomes can be secreted by almost all cell types.^9^ The biogenesis of exosomes is an extremely complex process (Figure 1).^10^, ^11^, ^12^ Their cargo molecules can promote numerous physiological and immunomodulatory processes by mediating communications between cells and organs. Engineered exosomes can also serve as effective vehicles for therapeutic drug delivery in vivo.^11^, ^13^, ^14^ Furthermore, the compositions, production rates and volumes of exosomes differ among various cellular origins,^15^, ^16^ and can be altered by various stimuli or pathological factors. As a result, exosomes hold diagnostic potential for diseases and can provide insights into disease progression.^11^

Compared to MSCs, MSC‐Exo possess several advantages, including their smaller size, greater safety, higher stability and strong biological effects. Moreover, MSC‐Exo can be sterilized through filtration for clinical applications.^17^, ^18^ However, current studies suggest that MSC‐Exo face several critical challenges in clinical translation that remain unresolved. For example, the spontaneous secretion of MSC‐Exo is insufficient to ensure an adequate quantity for clinical utility.^19^ Due to the heterogeneity of MSC‐Exo, there is no recognized, standardized method for large‐scale isolation and production.^20^, ^21^, ^22^ Additionally, there is a lack of suitable tracking and detecting methods in vivo after application of MSC‐Exo.^23^, ^24^, ^25^ Therefore, further exploration is needed to enhance the potential of MSC‐Exo.

Physical factors can be categorized into two aspects: one pertains to the physical characteristics of internal microenvironment, such as substrate stiffness and topography,^26^, ^27^, ^28^, ^29^ while the other involves external physical stimulations, including mechanical strain, ultrasound, laser, electrical stimulation and other stimuli.^30^, ^31^, ^32^, ^33^, ^34^ Although intense physical stimulation is a major factor contributing to tissue damage,^35^, ^36^ it can be widely used as a therapeutic approach in a variety of diseases if the parameters are properly adjusted.^37^, ^38^, ^39^, ^40^ Previous studies have demonstrated that exosomes are highly sensitive to physical factors.^41^, ^42^ Appropriate stimuli can induce beneficial changes in MSC‐Exo, thus enhancing their impact on the microenvironment. Faced with the challenges of MSC‐Exo in clinical translation, physical factors have recently gained broad application for the intervention and enhancement of exosomes.^19^, ^43^, ^44^, ^45^

On one side, the application of physical factors can mimic the dynamics physical microenvironment both in vivo and in vitro, aiding in the exploration of alterations in MSC‐Exo under different physical conditions.^46^, ^47^ On the other hand, physical factors can be regarded as technical methods for engineering MSC‐Exo.^19^, ^48^ In this context, we first provide a brief overview of the effects of physical stimuli on exosomes derived from various cell types. Then, we delved into recent literature regarding the impacts of different physical factors on MSC‐Exo, elucidating the influence of different physical factors and their related molecular mechanisms. Finally, we present deeper insights into MSC‐Exo‐based therapy.

The literature search strategy focused on key terms such as ‘Mesenchymal stem cells’, ‘Exosomes’ and ‘Physical factors’ in combination with specific physical factors such as ‘Ultrasound’, ‘Electrical stimulation’, ‘Light irradiation’, ‘Intrinsic physical properties’, ‘Ionizing radiation’, ‘Magnetic field’, ‘Mechanical forces’ and ‘Temperature’. Synonyms and alternative terms were also taken into consideration. Searches were conducted in academic databases, including PubMed, Scopus and Web of Science, to retrieve relevant articles. We aimed to include recent and highly cited articles in the field of mesenchymal stem cell‐derived exosomes and their applications in regenerative medicine (Figure S1).

MSCs can be isolated from a variety of tissues, including skin, dental pulp, neonatal tissues, muscle, adipose tissue, bone marrow and menstrual blood. Microvesicles (50–1000 nm) are released from MSCs through plasma membrane budding, while MSC‐Exo (40–160 nm) are derived through the endocytic pathway. To put it briefly, the process begins with the internalization of extracellular bioactive components and fluids through endocytosis, along with cell surface proteins. This leads to the formation of early‐sorting endosomes (ESEs) through the budding of plasma membrane. ESEs subsequently grow into late‐sorting endosomes (LSEs). Invagination within the LSEs generates intraluminal vesicles (ILVs), which ultimately assemble into multivesicular bodies (MVBs). The biogenesis of exosomes involves various proteins, including the Ras‐related protein GTPase (Rab),^49^ apoptosis‐linked gene 2‐interacting protein X (Alix)^50^ and numerous others.^11^ Finally, MVBs can be recycled by the cells through lysosomal degradation; or docked at the plasma membrane for the generation and release of exosomes. The released exosomes are nanoparticles enclosed by a lipid bilayer. Exosome surface proteins include tetraspanins, adhesion molecules, surface antigens and more. The cargo of exosomes contains a variety of biological components, including proteins, nucleic acids and lipids.^11^ Exosomes can carry different types of surface proteins and intracellular biomolecules, thereby inducing various biological responses. The distinct combinations of their sources, sizes and contents contribute to the high heterogeneity of exosomes.^11^

## 2 EFFECTS OF PHYSICAL FACTORS ON EXOSOMES

The initial inspiration to explore the impact of physical factors on exosomes came from the observation that blood cells release exosomes in response to fluid shear stress (FSS) within the bloodstream in vivo.^51^, ^52^ Subsequently, numerous studies have confirmed that both the production and biological characteristics of exosomes can be sensitively modulated through exposure to various physical stimuli.^19^, ^41^, ^48^ Additionally, physical factors have emerged as technical solutions for engineering exosomes and generating simulated nano‐vesicles.^53^, ^54^, ^55^ As a result, most of the processes in the biogenesis of exosomes, including generation, separation, migration, and engineering, can be powered by physical factors.^41^ Considering the limitations associated with clinical applications of exosomes, such as a short circulatory half‐life, uncontrolled biological functions, poor therapeutic targeting, heterogeneity and challenges in achieving standardized large‐scale production,^56^, ^57^, ^58^, ^59^ physical factors hold the potential to serve as effective tools for addressing these issues.

### 2.1 Effects of physical factors on the generation and secretion of exosomes

Several internal and external physical signals, including substrate stiffness^60^ and topography,^46^ shear stress,^61^, ^62^, ^63^ ultrasound,^64^, ^65^, ^66^ laser irradiation,^67^, ^68^ electrical stimulation,^69^, ^70^, ^71^ ionizing radiation^72^, ^73^ and others,^74^ have been shown to enhance the generation and secretion of exosomes. Guo et al. confirmed that applying mechanical stimuli to bioreactors tremendously increased the yield of MSC‐Exo, primarily mediated through the mechanosensitive protein, yes‐associated protein (YAP).^75^ This study emphasized the crucial role of physical factors in exosome production. Additionally, ultrasound and mechanical forces have been demonstrated to assist in the separation of cell membranes and the isolation of exosome‐like vesicles,^76^, ^77^, ^78^, ^79^ thereby boosting the production of artificial therapeutic exosomes. An interesting study carried out by Zhang et al.^54^ demonstrated that exosome‐mimetic vesicles generated using the method mentioned above and natural exosomes derived from MSCs exhibited highly consistent protein compositions and displayed comparable therapeutic efficacy in promoting wound healing.

### 2.2 Effects of physical factors on biological properties of exosomes

First, numerous studies have reported that diverse physical stimulations can alter the composition and biological properties of exosomes. Several researchers have provided evidence that the bioactivity and biological composition of exosomes are influenced after exposure to ionizing radiation.^72^, ^80^, ^81^, ^82^ In addition, FSS,^83^ microgravity,^84^ substrate stiffness,^60^ electrical stimulation^85^, ^86^ and ultrasound^65^ have been proved to have the capacity to influence the bioactive cargo of exosomes, resulting in modifications to their functional characteristics. Notably, a recent study proposed that further investigation is needed to determine the optimal settings of photobiomodulation (PBM) in the context of MSC‐Exo modification,^87^ while another study evidenced that light‐emitting diode irradiation suppressed the angiogenic capacity of exosomes secreted by uterine adenocarcinoma cells.^88^

Second, physical properties can also be introduced to exosomes to enhance their functionality. For instance, exosomes can be equipped with magnetic properties by loading magnetic nanoparticles. Following that, the targeted delivery path of exosomes can be controlled,^89^, ^90^ allowing exosomes to exert the effect of magnetic hyperthermia in vivo^91^ and facilitating the tracking of exosome biodistribution with magnetic labels.^92^, ^93^ Similarly, responsive exosome delivery and treatment systems can be developed for disease treatment by incorporating photosensitizers.^94^, ^95^, ^96^ Conversely, physical factors, such as mechanical stress,^97^ ultrasound^97^, ^98^ and electrical stimulation^55^, ^99^, ^100^ can also be employed as technical means to assist in the drug loading of exosomes. However, it should be noted that the safety of exosomes modified using these methods must be rigorously assessed to mitigate potential cytotoxicity.^98^, ^100^

Furthermore, freeze‐drying of exosomes using physical methods has been shown to preserve the biological activity of exosomes for an extended period of time.^101^, ^102^, ^103^ This innovation holds significant promise for the long‐term preservation and storage of exosomes, thus facilitating their clinical application.

### 2.3 Application of physical factors in the preparation of ‘nanoghosts’

‘Nanoghosts’ are regarded as another special type of exosome‐mimetic nanovesicles. Given the challenges posed by the heterogeneity and limited yield of exosomes, the stimulation of several physical factors (such as cavitation, extrusion and ultrasound) can be employed to initially disrupt cells. This process promotes the separation of the cell membrane while eliminating all cytosolic components. These engineered nanovesicles consist solely of typical membrane proteins, and are devoid of functional cytosolic proteins and nucleic acids.^41^, ^104^, ^105^, ^106^ These nanovesicles possess the advantageous characteristics such as biocompatibility, homogeneity and excellent drug delivery capacities.^41^, ^107^ Therefore, these particles have promising potential in clinical application, both in terms of their normalized production and their function as tissue‐specific drug carriers. This success can be attributed to the rational application of physical factors in tissue engineering.

## 3 EFFECTS OF DIFFERENT PHYSICAL FACTORS ON MSC‐Exo

**FIGURE 2:**
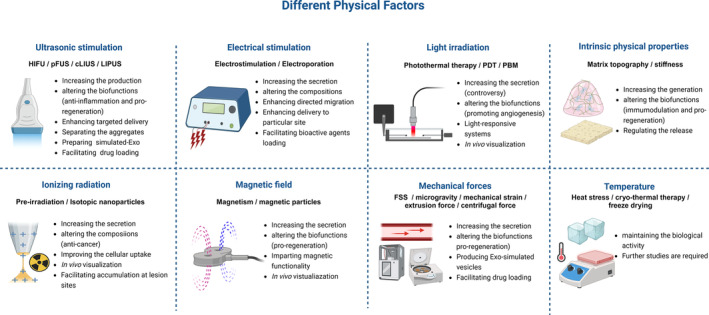
Application of different physical factors to MSC‐Exo. Physical factors with diverse modalities may contribute to different influences on MSC‐Exo.

Recent research efforts have predominantly centred on how different physical factors can increase secretion,^46^, ^108^ reduce heterogeneity,^109^ induce changes in biological behaviours^110^ and enhance the therapeutic potential of MSC‐Exo^111^, ^112^ (Table 1, Figure 2).

**TABLE 1:** The effects of physical stimulations on MSC‐Exo.

Physical stimulations		Effects	Biological effects	Cells	References
Ultrasound stimulation	LIPUS	Increasing the production		BMSCs	^39^, ^105^
	LIPUS	Altering the biological functions	Anti‐inflammatory effects	BMSCs, DMSCs	^28^, ^105^
	LIPUS		Promoting cartilage regeneration	BMSCs	^39^, ^119^
	LIPUS		Promoting osteogenic differentiation	DMSCs	^28^
	pFUS		Improving the treatment of acute kidney injury	BMSCs	^127^, ^128^
	pFUS	Enhancing the targeted delivery in vivo		Endothelial progenitor cells	^104^
	pFUS	Promoting the separation of MSC‐Exo aggregates		ADSCs	^129^
	Ultrasonication	Preparing simulated MSC‐Exo		UC‐MSCs	^103^
	Ultrasonication	Facilitating drug loading into MSC‐Exo		Macrophages	^92^
Electrical stimulation	Electric stimulation	Increasing the production		Cardiac MSCs	^136^
		Altering the biological functions	Enhancing the cardioprotective effects	Cardiac MSCs	^136^
			Increasing the concentrations of BMP‐2 in the medium	ADSCs	^138^
	In vivo electroporation	Enhancing the targeting efficacy	Improving the retinal delivery	ADSCs	^55^
	Direct current		Improving the directed delivery	BMSCs	^140^
	Electroporation	Improving the encapsulation of bioactive substances into MSC‐Exo		BMSCs, UC‐MSCs	^100^, ^141^, ^142^, ^143^, ^144^, ^145^, ^146^
Light irradiation	PBM (low‐level laser irradiation)	Only a slight change in the number and functions of MSC‐Exo		PDLSCs, ADSCs	^87^
	Blue light illumination (455 nm)	Enhancing the proangiogenic ability	Altering the biological contents	UC‐MSCs	^67^
	Visible light irradiation	Delivery system for photosensitizers	Promoting the injection‐free delivery	BMSCs	^94^
	PDT (650 nm)		Enhancing the efficacy of PDT	C3H/10T1/2	^153^
	Near‐infrared laser		Triggering the controlled release	BMSCs	^154^
	Blue light irradiation		Promoting loading drugs	UC‐MSCs	^155^
	Fluorescent dyes		Realizing the visualization of MSC‐Exo in vivo	UC‐MSCs, BMSCs	^23^, ^92^
Intrinsic physical properties	Substrate topography	Increasing both the quantity and angiogenic ability of MSC‐Exo		ADSCs	^156^
		Exerting immunomodulatory effects	Inducing the M1 polarization of macrophages	BMSCs	^157^
		Enhancing osseointegration	Increasing the generation and extracellular secretion of MSC‐Exo	BMSCs	^46^
	The stiffness of the extracellular matrix or exosome‐loaded hydrogels	Affecting the MSC secretome		BMSCs	^161^
		Regulating the release of MSC‐Exo		UC‐MSCs	^162^
Ionizing radiation	X‐Ray irradiation (2 Gy)	Increasing the secretion		UC‐MSCs	^166^, ^167^
		Inducing the biological activation	Altering the biological compositions	UC‐MSCs	^167^
	γ‐Ray irradiation (8 Gy)	Improving the cellular uptake of MSC‐Exo	Increasing the formation of CD29/CD81 complexes on the treated cell surface	BMSCs	^171^
	Radioactive labels	Enabling biological imaging and noninvasive tracking of MSC‐Exo in vivo		Cord blood MSCs, BMSCs	^172^, ^173^, ^174^
Magnetic field	Magnetic nanoparticles combined with magnetic field	Increasing the secretion		BMSCs	^108^, ^176^
		Promoting greater bone regeneration	Improving osteogenesis and angiogenesis	BMSCs	^176^
		Improving the efficacy of targeted therapy	Promoting the treatment of ischemic stroke	BMSCs	^89^
			Promoting cardiac function recovery	BMSCs	^112^
			Promoting wound healing	UC‐MSCs	^177^
			Enhancing skin retention and hair follicle growth	ADSCs	^179^
		Enabling real‐time monitoring of the biodistribution in vivo		BMSCs, UC‐MSCs	^23^, ^92^
		Inducing magnetic hyperthermia	Facilitating targeted tumour cell ablation	BMSCs, UC‐MSCs	^91^
	Magnetic 3D bioassembly platforms	Promoting skin repair		DPSCs	^178^
Mechanical forces	Mechanical strain	Inducing both the secretion and the myogenic function		Muscle progenitor cells	^182^
	Extrusion pressure, centrifugal force	Encouraging the preparation of MSC‐Exo‐like nanovesicles		ADSCs	^183^

Abbreviations: ADSCs, adipose‐derived mesenchymal stem cells; BMP‐2, bone morphogenetic protein‐2; BMSCs, bone marrow mesenchymal stem cells; DMSCs, human dental mesenchymal stem cells; DPSCs: dental pulp stem cells; PDLSCs, periodontal ligament stem cells; PBM, photobiomodulation therapy; PDT, photodynamic therapy; UC‐MSCs, human umbilical cord mesenchymal stem cells.

### 3.1 Ultrasonic stimulation

Currently, ultrasound has been widely applied as a safe and non‐invasive intervention in the medical field.^113^ In brief, ultrasound can be broadly categorized into shockwaves, therapeutic ultrasound, and diagnostic ultrasound. Within therapeutic ultrasound, there are various modalities, including high‐intensity focused ultrasound (HIFU), pulsed focused ultrasound (pFUS), continuous low‐intensity ultrasound (cLIUS) and low‐intensity pulsed ultrasound (LIPUS).^114^

Liu et al.^114^ provided an overview of the enhanced effectiveness of ultrasound in MSC‐based therapies, describing how ultrasound can influence the biological traits of MSCs. A systemic review also reported that LIPUS could modulate the proliferation and directional differentiation of MSCs.^115^ Furthermore, LIPUS has been also verified to strengthen the potential for tissue repair and regeneration in various diseases.^116^, ^117^, ^118^, ^119^, ^120^ The mechanisms underlying these effects may be related to the impact of ultrasound on MSC‐Exo. In the case of MSC‐Exo, the effects of ultrasound can be categorized into six key aspects: increasing production, altering biological functions, enhancing targeted delivery in vivo, promoting the separation of MSC‐Exo aggregates, generating simulated MSC‐Exo and facilitating drug loading into MSC‐Exo.

#### 3.1.1 Ultrasound in tissue engineering

When considering the characterization of MSC‐Exo, the application of appropriate low‐intensity ultrasound can assist in disaggregating MSC‐Exo, thus facilitating their utilization in experimental and clinical applications. However, it is essential to finely adjust the parameters to avoid exosome heterogeneity following treatment.^121^

Furthermore, ultrasound can serve as a valuable technical tool in tissue engineering for the preparation of MSC‐Exo‐mimetic particles^109^ and for aiding in the loading of therapeutic agents into macrophage‐derived exosomes.^98^ It seems that there have been limited studies exploring the application of ultrasonication in drug loading into MSC‐Exo. These capabilities contribute to the large‐scale production of standardized, bio‐functional MSC‐Exo.

#### 3.1.2 The impact of LIPUS on MSC‐Exo

Recent studies have confirmed the ability of LIPUS to stimulate the secretion of MSC‐Exo.^42^, ^111^ The most recent research has reported that LIPUS can significantly reduce the expression of pro‐inflammatory cytokines and contribute to the modulation of the immune microenvironment in vivo.^122^, ^123^, ^124^, ^125^ In alignment with these findings, the application of LIPUS to MSC‐Exo reinforces their anti‐inflammatory effects via altering the compositions of MSC‐Exo.^31^, ^111^

Additionally, LIPUS has been acknowledged as a potent intervention to promote cartilage regeneration and osteogenic differentiation mediated by MSC‐Exo.^31^, ^42^, ^126^ Besides, LIPUS could accelerate nerve injury repair.^127^, ^128^ Interestingly, MSC‐Exo also exhibit robust therapeutic potential in neurological diseases.^129^, ^130^, ^131^ LIPUS has been confirmed as an effective intervention to augment the differentiation of neural stem cells into neural cells^132^ and improve MSC‐based therapies for functional nerve recovery.^116^, ^133^However, it is worth noting that there is limited scientific exploration regarding the effects of LIPUS on MSC‐Exo in neural tissue engineering, which warrants further investigation in the future.

#### 3.1.3 The combination of pFUS with MSC‐Exo

As another form of therapeutic ultrasound, the combination of pFUS and MSC‐Exo has enhanced the anti‐inflammatory and regenerative efficacy of exosomes in the treatment of acute kidney injury.^134^, ^135^ Furthermore, when administered with appropriate parameters, pFUS can facilitate the targeted delivery of MSC‐Exo to the stroke region without causing damage to normal brain structures.^110^ A clinical study utilizing concurrent transcranial ultrasound to enhance the exosome delivery in the brain is currently underway (NCT04202770).

### 3.2 Electrical stimulation

Similar to other physical factors, electrical stimulation greatly increases the secretion of exosomes from cardiac‐derived MSCs.^136^ Previous studies have confirmed that electrical stimulation can exert a notable impact on the biological properties of MSCs, particularly their directional differentiation potential, such as osteogenic differentiation^137^, ^138^ and neurogenic differentiation.^139^ These effects may be attributed to changes in MSC‐Exo induced by electrical stimulation, as evidenced by differences in the bioactive substances present in the conditioned medium of MSCs following electrical stimulation.^138^ However, specific studies on the application of electrical stimulation to MSC‐Exo remain somewhat limited. A recent study performed by Zhang et al.^136^ found that electrical stimulation increased the function of cardioprotective proteins in cardiac MSC‐Exo.

Electrical factors can physically induce the directed migration of MSCs in an electric field, consequently enhancing the targeting efficacy of MSC‐Exo.^140^ Moreover, electroporation optimized the delivery of MSC‐Exo to specific sites in vivo, such as the eyeball.^55^ Electroporation was also a widely‐used method for encapsulating bioactive substances into MSC‐Exo.^100^, ^141^, ^142^, ^143^, ^144^, ^145^, ^146^ With the homing feature to pathological sites of MSC‐Exo, the administration of electroporation significantly enhances the targeting efficacy of therapeutic drugs.

### 3.3 Light irradiation

As a form of physical treatment, phototherapy consists of photothermal therapy (PTT), photodynamic therapy (PDT), photobiomodulation therapy (PBM) and other modalities.^147^, ^148^ Studies have demonstrated that low‐level laser therapy possesses therapeutic potential, including anti‐inflammatory and regenerative effects.^149^ Moreover, previous research has indicated the positive effects of PBM on enhancing the viability and proliferation of MSCs.^150^

However, there is a relatively limited number of studies exploring the impact of laser irradiation on MSC‐Exo. Low‐intensity laser irradiation has been evidenced to significantly increase the secretion of exosomes derived from endothelial cells^151^ and dermal papilla cells.^152^ Conversely, after the lowest‐intensity laser irradiation, there was only a slight change in the number of exosomes secreted by MSC without statistical significance, as well as the functions of MSC‐Exo.^87^ This suggests that further investigation is needed to determine the optimal settings of PBM for MSC‐Exo, as the intensity of laser irradiation may be too low to generate enough energy to stimulate improvements in MSC‐Exo. Another study validated the profitable influence of blue light on MSC‐Exo, particularly in boosting angiogenesis,^67^ which was attributed to the alterations of miRNAs in MSC‐Exo.

Additionally, MSC‐Exo is considered a novel and advantageous delivery system for photosensitizers. Combined with the ‘homing effect’ of MSC‐Exo and the noninvasive property of PDT, the clinical effectiveness of MSC‐Exo has been greatly improved.^94^, ^153^ Based on the light‐responsive system, light irradiation can not only trigger the controlled release of engineered MSC‐Exo,^154^ but also promote the loading of drugs into MSC‐Exo.^155^ Furthermore, with the aid of an in vitro imaging system, fluorescent dye‐labelled MSC‐Exo have made it possible to visualize MSC‐Exo in vivo.^23^, ^92^ This advancement contributes to a deeper understanding of the trajectory of MSC‐Exo as they enter our body.

### 3.4 Intrinsic physical properties (substrate topography and stiffness)

MSC‐Exo are highly sensitive to the intrinsic physical properties of their microenvironment. For example, compared to a planar matrix, substrate topography with micro‐grooves enhances both the quantity and angiogenic ability of MSC‐Exo.^156^ Recently, a study conducted by Zhang et al.^46^ provided evidence that the improved osteogenic effect of micro/nano‐textured layered titanium topography could be ascribed to increased generation and elevated extracellular secretion of MSC‐Exo, which was facilitated by the activation of the RAB27B and SMPD3 pathways. Conversely, another study demonstrated that the TiO_2_ nanoporous topography promoted MSC‐Exo to play an immunomodulatory role by inducing the M1 polarization of macrophages, potentially having a detrimental impact on the osteogenesis process.^157^

Furthermore, the stiffness of the extracellular matrix (ECM) also mattered. Excessive ECM deposition in the tumour microenvironment may lead to an increase in substrate hardness, thereby promoting the secretion and modulating the biological behaviours of exosomes derived from cancer cells.^60^, ^158^ Although there were still few special studies on the effect of substrate stiffness on MSC‐Exo, several previous studies have shown that the functional characteristics, including proliferation, differentiation and paracrine activity, are influenced to some extent by ECM stiffness.^159^, ^160^, ^161^ In particular, Vilar et al.^161^ confirmed that the substrate stiffness affects the MSC secretome to a large degree, indicating that the impact of ECM stiffness on MSCs can be explained by alterations in MSC‐Exo. Additionally, a study suggested that the stiffness of hydrogels can regulate the release of MSC‐Exo in tissue engineering, consequently affecting the therapeutic effect of MSC‐Exo.^162^

### 3.5 Ionizing radiation

MSCs are sensitive to direct ionizing radiation‐induced effects, but less sensitive to bystander effects caused by ionizing radiation.^163^ The bystander effect refers to the impact of ionizing radiation on the paracrine secretome of irradiated cells, leading to delayed and distant effects in non‐radiated cells.^164^ Consequently, combining radiotherapy with MSC‐based therapy is a feasible approach.

An increasing number of studies have demonstrated the significant influence of ionizing radiation on the release and bio‐properties of exosomes derived from keratinocytes,^81^ cancer cells^72^, ^80^, ^165^ and other sources.^82^ Consistently, ionizing radiation has also been shown to increase the secretion of MSC‐Exo.^166^, ^167^ Second, ionizing radiation can induce the biological activation of MSCs, especially at a pre‐irradiation of 2Gy, resulting in quantitative and qualitative changes in MSC‐Exo.^167^ Particularly, the presence of Annexin A1 (ANXA1) in the exosomes secreted from pre‐irradiated MSCs has been confirmed to be unique. ANXA1 has been reported to be crucially involved in extensive processes beyond immune responses in vivo.^168^ de Araújo Farias et al.^169^ suggested that conditioned media derived from irradiated MSCs were cytotoxic to tumour cells, possibly due to alterations in the biological composition of MSC‐Exo induced by ionizing radiation.^167^

Radiation‐induced changes in the composition of serum exosomes could potentially serve as diagnostic biomarkers for assessing a history of radiation exposure.^170^ Similarly, investigating whether radiation‐related biomarkers of MSC‐Exo could help assess the curative effect of exosomes is an intriguing research direction. Ionizing radiation is recognized to improve the cellular uptake of MSC‐Exo through increasing the formation of CD29/CD81 complexes on the cell surface.^171^ Additionally, the application of gold nanoparticles for labelling MSC‐Exo enabled biological imaging and non‐invasive tracking of MSC‐Exo in vivo.^172^, ^173^, ^174^ It is noteworthy that, given MSC‐Exo's ability to traverse the blood–brain barrier, incorporating isotopic nanoparticles could enable in vivo neuroimaging and enhance the accumulation of MSC‐Exo at lesion sites, potentially augmenting the therapeutic efficacy of MSC‐Exo for neurological disorders.^175^

### 3.6 Magnetic field

It has been reported that the application of an electromagnetic field increases the secretion of MSC‐Exo and heightens their ability to induce osteogenic and angiogenic differentiation.^108^, ^176^

In the context of tissue engineering, the introduction of magnetic particles to MSC‐Exo endows MSC‐Exo with magnetic functionality. For example, MSC‐Exo incorporated with iron oxide nanoparticles can markedly improve the efficacy of targeted therapy for ischemic lesions.^89^ Magnetic MSC‐Exo have also found beneficial effects on cardiac function recovery,^112^ skin repair and wound healing,^177^, ^178^ maintenance of skin rejuvenation^179^ and promotion of hair growth.^179^ Except that, magnetic MSC‐Exo can induce magnetic hyperthermia while accurately targeting tumour cells.^91^

Leveraging magnetism, MSC‐Exo can be equipped with particular physical markers, enabling real‐time monitoring of their biodistribution in vivo using magnetic resonance imaging (MRI) technology.^23^, ^92^

### 3.7 Mechanical forces

Mechanical forces include FSS, microgravity, mechanical strain, extrusion pressure, centrifugal force and so on.^26^ Studies have demonstrated that FSS and microgravity can regulate the constituents of exosomes derived from endothelial cells^83^ and cancer cells.^84^, ^180^, ^181^ However, the impact of FSS and microgravity on MSC‐Exo remains relatively unexplored.

A clinical trial purposed to explore the effects of resistance exercise on exosomes derived from skeletal muscle in improving adipose metabolism has been conducted by John McCarthy's team from the United States (NCT04500769). Similarly, a study performed by Mullen et al.^182^ reported that the application of mechanical strain induced the secretion of exosomes produced from muscle progenitor cells and increased their myogenic function via modulating their miRNA contents. Such studies may contribute to a better understanding of the mechanisms by which exercise affects the body on a cellular level.

Furthermore, similar to ultrasound, extrusion pressure and centrifugal force can encourage the preparation of MSC‐Exo‐like nanovesicles^183^ and provide assistance in the development of targeted delivery systems using MSC‐Exo as drug vehicles.^77^

### 3.8 Temperature

Accumulated studies have reported that heat stress exerted significant effects on both the volume and contents of exosomes produced by various sources, including cancer cells, immune cells and blood.^184^, ^185^, ^186^ Cryo‐thermal therapy can induce the release of serum‐derived exosomes and enhance their immunoregulation ability.^187^ However, further studies are required to determine whether alterations occur in the secretion and biological functions of MSC‐Exo under varying temperature. Additionally, exploring whether pre‐treatment at different temperatures can be considered a strategy to enhance the resilience of MSC‐Exo in extreme environments is a promising avenue for future research.

## 4 MECHANISMS OF PHYSICAL FACTORS AFFECTING EXOSOMES

As we mentioned above, FSS in the bloodstream greatly increases the release of exosomes derived from blood cells.^52^ The mechanisms by which mechanical forces affect exosome release may be caused by the regulation of membrane tension. The altered membrane tension regulates the membrane area through passive morphological transformation of the membrane, including membrane folding, endocytosis, and the formation of outward membrane tubules due to lateral compression.^188^ In addition, when increased membrane tension leads to the rupture of the cell membrane, elevated intracellular Ca^2+^ triggers exocytosis through the coordination with the Rab family^189^ and promotes the degradation of cytoskeleton induced by Ca^2+^‐dependent proteases.^190^ Physical stimuli, such as ultrasound, mechanical strain and magnetic force, can also facilitate the secretion of exosomes by stimulating autophagy through increased expression of Beclin‐1 and LC3,^42^, ^108^ modulation of the movement of actin filaments,^182^ and up‐regulation of proteins related to exosome biogenesis and docking (including the Rab family and Alix).^108^, ^122^, ^156^ It has been reported that electrical stimulation boosts the production of ceramide through increased expression of neutral sphingomyelinase 2 (nSMase2), thus promoting the secretion of exosomes under stress.^136^

**FIGURE 3:**
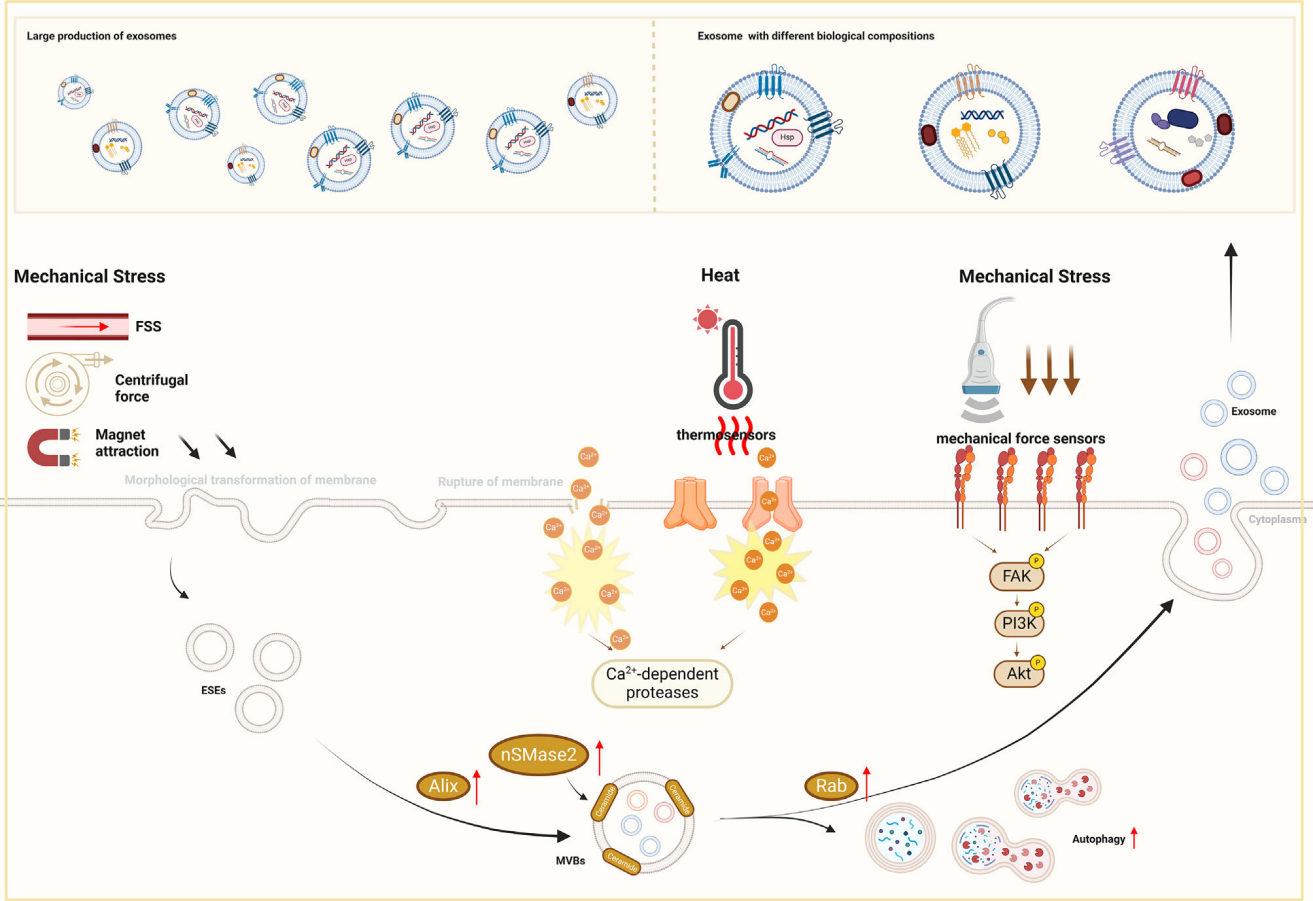
Mechanisms of physical factors affecting exosomes. The known and possible mechanisms of physical factors affecting exosomes. Akt, protein kinase B; Alix, (apoptosis‐linked gene 2) ALG‐2‐interacting protein X; ESEs, early‐sorting endosomes; FAK, Focal adhesion kinase; FSS, fluid shear stress; MVBs, multivesicular bodies; nSMase2, neutral sphingomyelinase2; Pi3k, phosphatidylinositol‐3‐kinase.

On the other hand, physical factors including ultrasonication of different intensities and magnetic forces can exert distinct physical effects, such as thermal effects and mechanical effects.^122^ Correspondingly, thermal effects may induce conformational changes in certain temperature‐dependent proteins^191^ and the shock waves or attraction generated by physical stimuli may activate mechanical sensors (such as integrins).^192^ The effects imposed by physical stimulations can activate various downstream pathways via the sensation of sensors, such as the TLR4–MyD88 pathway^193^ and the PI3K/Akt pathway.^192^ Therefore, exosomes with different biological compositions, including micro‐RNA (miRNA),^67^ growth factors and cytokines^156^ and functional proteins^136^ would be secreted by MSCs then. Consequently, these modifications influence the biological functions of exosomes, such as anti‐inflammation and pro‐regeneration (Figure 3).

## 5 POTENTIALITY OF PHYSICAL STIMULI‐AFFECTED MSC‐Exo IN CLINICAL APPLICATION

Based on corresponding research, various physical factors can be applied to modify MSC‐Exo for specific clinical purposes in the future. Alternatively, the in vivo or in vitro environment can be imitated to explore changes in MSC‐Exo under various states.

In terms of treatment, for example, a patent P201500022 registered in 2014 focused on the activation of MSCs induced by pre‐irradiation. This affirmed that ionizing radiation‐affected MSC‐Exo could improve the radiotherapy outcomes for tumours and potential metastatic foci without toxicity, offering a novel and effective strategy for cancer therapy.^194^ Considering the anti‐inflammatory, pro‐regenerative and delivery‐enhanced effects of ultrasound on MSC‐Exo, several inflammatory or locally dysfunctional diseases may benefit from this combined therapy. Neurological Associates of West Los Angeles has also conducted a clinical study to evaluate the safety and efficacy of exosome combined with focused ultrasound in patients with depression, anxiety, and neurodegenerative dementia since 2019 (NCT04202770). Additionally, employing physical stimuli as a specific scale‐up strategy could increase the yield of both MSC‐Exo and exosome‐mimetic vesicles,^78^, ^105^ so as to meet the requirement for clinical treatment. By summarizing the diverse effects of physical factors on MSC‐Exo, the advantageous application area of physical stimuli‐modified MSC‐Exo could be identified.

To avoid pathological states, physical stimulations can be applied to simulate the physiological microenvironment. MSC‐Exo pre‐treated with such stimuli could be induced to have a specific therapeutic impact. For instance, the application of electrical stimulation has been reported to increase the composition of cardioprotective proteins in cardiac MSC‐Exo^136^; therefore, such MSC‐Exo could then be considered for use in cardiovascular diseases. By changing the matrix topography where MSC‐Exo are generated to simulate the 3D microenvironment in the osteogenesis process,^46^ the osteogenic function of MSC‐Exo could be improved. Such MSC‐Exo could then play a more powerful role in refractory bone defects. Broadly, physical stimuli can also be employed to mimic special physical environments (extreme temperature, weightlessness, vibration, etc.) to trigger compensatory effects of MSC‐Exo, thereby increasing the stability of MSC‐Exo when applied in these conditions.

Moreover, MSC‐Exo and exosome‐mimetic vesicles produced through physical methods can serve as nanocarriers for drugs and bioactive molecules.^78^, ^105^, ^107^, ^195^ Several clinical trials applying exosomes encapsulated with super‐repressor IκBα (NCT05843799), siRNA against KrasG12D (NCT03608631) and Ldlr mRNA (NCT05043181) have been carried out for the treatment of specific diseases, indicating the potential clinical effect of MSC‐Exo as carriers in vivo. However, the sources of exosomes and the production methods of MSC‐Exo were not detailed in these trials. Similarly, single or multiple physical agents can also be loaded into MSC‐Exo to equip them with extra physical properties simultaneously.^196^, ^197^ In addition to physical factors‐induced treatment effects (such as photothermal effect and magnetothermal effect), physically‐triggered MSC‐Exo could realize in vivo imaging, photosensitive response release and other special physical effects as well. In these aspects, the therapeutic potential of engineering MSC‐Exo is considerably significant; however, relevant clinical trials are still lacking.

Currently, more than 40 clinical trials have been registered on clinicaltrials.gov to assess the safety and efficacy of MSC‐Exo for the treatment of diverse diseases. However, there are only a few clinical trials applying MSC‐Exo modified by physical stimuli or combined with physical factors. In the long term, the incorporation of physical factors into both the yield and enhancement of MSC‐Exo is poised to enhance the potential of MSC‐Exo‐based therapy for regenerative medicine, offering a greater range of therapeutic possibilities.

## 6 DISCUSSION

Research in this field has the potential to significantly advance our understanding of disease pathogenesis and progression while offering promising avenues for MSC‐Exo‐based therapies for refractory diseases. These studies can be particularly valuable for individuals working in unique environments like astronauts or polar expeditions, where the effects of physical factors on MSC‐Exo could have practical applications. The influence of physical factors on MSC‐Exo extends beyond just the quality and quantity of exosomes, it encompasses a series of biological processes, from the formation of exosome‐like particles to their delivery in vivo and preservation in vitro.

However, the effects of physical stimulations on MSC‐Exo have primarily been observed at a superficial level, typically focusing on the alterations of secretion and compositions of exosomes. Further research is imperative to unravel the molecular mechanisms underlying the specific responses of MSC‐Exo to various physical stimuli. We could keep a watchful eye on how physical stimuli activate different cellular sensors and the downstream pathways they trigger. Additionally, investigating how physical stimulations affect the biogenesis of MSC‐Exo, including endocytosis, generation, docking of multivesicular bodies (MVBs) and exocytosis, is also a worthy avenue of in‐depth research.

In‐depth exploration must also consider that physical stimulations can initiate complex chain reactions. For example, generally, HIFU always affects the microenvironment through a thermal effect,^198^ rather than a mechanical one. Thus, it is essential to determine which physical factors genuinely contribute to altering exosome properties and not just examine surface‐level outcomes. Physical factors may act simultaneously, successively or singly, and different settings or combinations of factors can have varying effects. A broad understanding of the combined application of different physical factors is critical for maximum clinical and societal benefits. MSC‐Exo‐based therapy holds immense potential in regenerative medicine, providing hope for treating a wide range of challenging diseases and injuries. Therefore, the capability to precisely regulate MSC‐Exo secretion and biological behaviour using physical factors is of paramount importance.

Furthermore, heterogeneity has been observed under different parameters of physical stimuli in various articles. Consequently, further studies are imperative to investigate the impacts of diverse settings of physical factors on the yield, safety and efficacy of MSC‐Exo. While attention is given to the impact of physical factors on MSC‐Exo production, the quality of MSC‐Exo also needed to be detected. Increased secretion of exosomes does not necessarily translate to improved biocompatibility, safety, and therapeutic potential.^199^, ^200^ Hence, parameters should be fine‐tuned to establish the optimal physical stimulation paradigm of MSC‐Exo under different conditions. For example, bioreactors designed for large‐scale production of MSC‐Exo, offer precise control over the culture environment, including physical factors like temperature and mechanical stimuli. The data from studies in this field exactly serve as the foundation for setting different parameters in bioreactors.

## 7 CONCLUSION AND PROSPECTIVE

In conclusion, physical factors have been found to play a crucial role in influencing both the secretion and biological properties of MSC‐Exo. This article has provided a comprehensive overview of the effects of various physical factors on MSC‐Exo, highlighting their role in enhancing the exosome generation and secretion, the modulation of exosome biological properties and the development of ‘nanoghosts’. Different physical stimulations can not only trigger diverse bioactive effects on MSC‐Exo through different mechanisms, but also consistently augment the production of MSC‐Exo.

Most of the physical factors, such as ultrasound, electric stimulation and ionizing radiation, can increase the secretion and promote the mass production of MSC‐Exo or MSC‐Exo‐simulated nanoparticles. In terms of biofunctions, different physical factors can induce distinct biological processed through altering the components (including proteins and miRNAs) of MSC‐Exo or imparting specific physical properties to trigger physical effects (such as magnetic properties). Combined with the regulation of physical stimuli, MSC‐Exo possesses the potential for broader clinical application.

However, till today, research on physically modulated MSC‐Exo is still in its early stages, both in basic research and clinical trials. Several critical aspects, including the underlying molecular mechanisms, standardization of mass production, optimization of physical stimulus parameters, assessment of clinical effectiveness and safety in clinical applications, require further exploration and validation. Relevant studies in this field remain relatively limited.

Despite the challenges, the prospects for the future of MSC‐Exo‐based therapy are promising. The application of physical factors has the potential to facilitate the large‐scale production of standardized therapeutic MSC‐Exo, bringing us closer to its integration into clinical practice. As research in this area continues to evolve, we can anticipate significant advancements in the field of regenerative medicine.

## AUTHOR CONTRIBUTIONS

All authors contributed to the review conception. Dan Wu, Xiansheng Zhao and Jiaheng Xie drafted the manuscript and finished the drawing of figures. Dan Wu and Xiansheng Zhao contributed equally to this work. Ruoyue Yuan, Yue Li, Quyang Yang, Xiujun Cheng, Changyue Wu, Jinyan Wu and Ningwen Zhu participated in the paper modification. All authors read and approved the final manuscript.

## CONFLICT OF INTEREST STATEMENT

All authors declared no conflicts of interest.

## Supporting information


**Figure S1.** The flowchart of the search strategy.
