# Supplementary figures and images for: Physical modulation of mesenchymal stem cell exosomes: A new perspective for regenerative medicine
Source: Cell Prolif. 2024 Mar 10;57(8):e13630. doi: 10.1111/cpr.13630 (PMC11294442; doi:10.1111/cpr.13630)

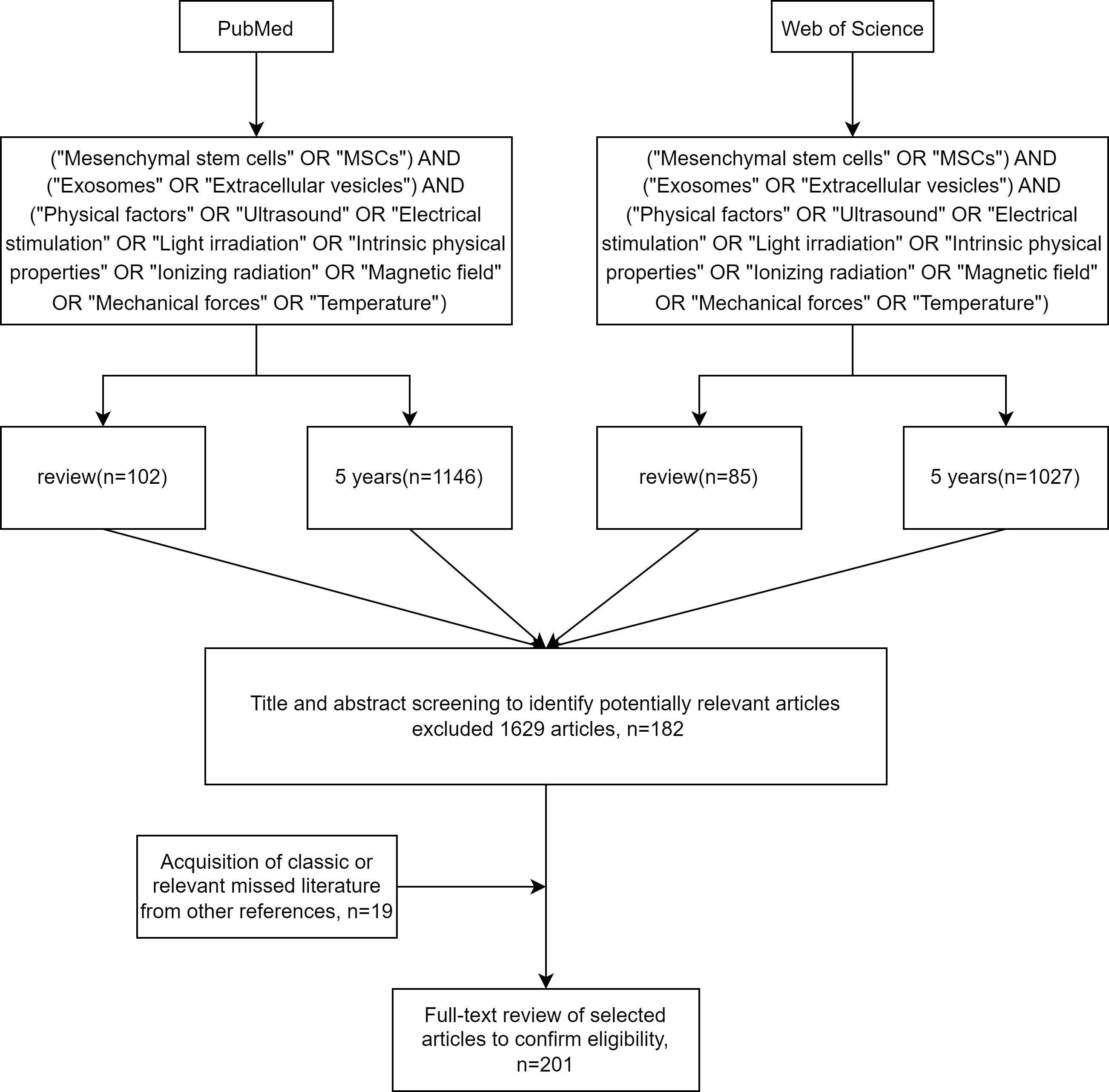

Supplement: Supplementary file 1 — Figure S1. The flowchart of the search strategy. [file CPR-57-e13630-s001.png]
